# Effect of Bamboo Essential Oil on the Oxidative Stability, Microbial Attributes and Sensory Quality of Chicken Meatballs

**DOI:** 10.3390/foods12010218

**Published:** 2023-01-03

**Authors:** Jyotishka Kumar Das, Niloy Chatterjee, Srija Pal, Pramod Kumar Nanda, Annada Das, Ligen Das, Pubali Dhar, Arun K. Das

**Affiliations:** 1 Eastern Regional Station, ICAR-Indian Veterinary Research Institute, 37 Belgachia Road, Kolkata 700 037, India; 2 Laboratory of Food Science and Technology, Food and Nutrition Division, University of Calcutta, 20B, Judges Court Road, Alipore, Kolkata 700 027, India; 3 Department of Livestock Products Technology, West Bengal University of Animal and Fishery Sciences, Kolkata 700 037, India; 4 National Food Laboratory, 3, Kyd Street, Taltala, Kolkata 700 016, India

**Keywords:** bamboo essential oil, antimicrobial, antioxidant, meatballs, food quality

## Abstract

This study explores the efficacy of bamboo essential oil (BEO) incorporated at 15 ppm (T1, BEO-I) and 30 ppm (T2, BEO-II) on the overall physicochemical and oxidative stability, microbial deterioration, and sensory acceptability of meatballs stored for 20 days under refrigerated conditions. Analysis of various parameters, including physicochemical quality, color (CIE L*, CIE a* and CIE b*), generation of oxidative products (TBARS), microbial growth, and sensory acceptability of meatballs were evaluated at 5-day intervals. In addition, the total phenolics and flavonoid content of BEO were estimated, and fatty acids were determined by Gas chromatography (GC.) To gain insights into the biological activities of the BEO, antioxidant assays were determined in vitro using various methods. The antibacterial activity of BEO was also evaluated against Gram-positive (*Staphylococcus aureus* and *Bacillus subtilis*) and Gram-negative (*Vibrio cholera*, *Salmonella* Typhimurium, *Shigella flexneri*, *Proteus vulgaris*, *Escherichia coli* and *Klebsiella pneumoniae*) bacterial strains. The BEO contained a good quantity of total phenolics and flavonoids. In addition, the oil exhibited very potent antioxidant activity scavenging reactive oxygen and other such species, effectively showing IC_50_ at a very minimal concentration. Further, the BEO exhibited a strong antibacterial effect with MICs within 2 µL and MBCs from 5 to 7 µL for Gram-positive as well as Gram-negative bacteria, respectively. At both the concentrations used, BEO did not show any negative effect on the color of cooked meatballs but rather increased the microbiological and oxidative stability during the overall storage period. Meatballs treated with BEO had considerably reduced oxidative changes in terms of TBARS levels compared to the control. The total viable microbial count was lowest in BEO-treated meatballs and the highest in control. Both control and treated meatballs had a desirable flavor and good acceptability. The sensory attributes and aroma of treated meatballs were better and acceptable during the storage study, whereas the control samples were disliked by the panelists on 15th day. From this study, it can be concluded that bamboo essential oil could be used as a benign and non-toxic preservative to improve the quality and shelf life of cooked meatballs stored under refrigerated conditions.

## 1. Introduction

Meat and other animal products are a rich source of protein, a variety of essential amino acids, and sufficient amounts of micronutrients, including minerals and vitamins. However, a number of attributes, such as variations in pH, water activity, temperature, light etc., create an environment that is conducive to oxidative reactions and microbial development affecting the shelf-life of products during storage. The microorganisms responsible for the spoilage of meat and meat products produce toxins, slime, undesirable flavors, off odors and colors, which decrease the quality and acceptability of the products [1]. Besides microbial contamination and spoilage, the most common form of chemical changes in meat products is due to oxidative (lipid and protein) changes that affect the meat constituents, such as proteins, fats, vitamins and pigments [2,3,4]. Again, lipid oxidation in food products negatively influences the functional properties, such as water-holding capacity, solubility of the protein, and even lowers the bioavailability of some major nutrients in physiological milieus. Further, lipid oxidation accelerates the production of off-odor (rancidity), off-flavor, changes the hue and consistency of meat and meat products, thereby deteriorating its quality [5,6]. The changes in sensory characteristics (color, flavor, odor, and texture) make meat products less attractive and reduce the rate of purchase and acceptability by consumers. Likewise, the oxidative changes of proteins reduce the water-holding capacity of protein, resulting in lower protein extractability and higher moisture loss during cooking [7]. The increase in meat dryness and low protein extraction lowers the binding capacity and emulsion stability of meat, especially when used for product development [4]. The control of microbial spoilage and oxidative processes of fresh as well as cooked meat is, therefore, important from the quality as well as safety point of view [8]. To overcome this problem, food manufacturers are using preservatives (food additives) having both antioxidants and antimicrobial activity to reduce oxidative changes, control microbial growth and extend the shelf-life of animal food products.

Essential oils from various plant sources, such as stems, fruits, roots, and leaves, are reported to be very effective for the preservation of meat and meat products. This is due to the existence of minute amounts of terpenoid fatty acids and phenolic components in essential oils that are said to have both bactericidal potential and antioxidant capability [9]. Again, the lipophilic characteristic of certain essential oils accounts for their antimicrobial properties. Thus, essential oils can be the best natural green alternatives to synthetic preservatives, such as butylated hydroxytoluene (BHT), sodium benzoate and butylated hydroxyanisole (BHA), since they have an equivalent or better impact on lowering lipid oxidation and bacterial growth [9,10]. Many essential oils, such as thyme, rosemary, oregano, basil, ginger, clove etc., are being used as antioxidant and antimicrobial additives in fresh, minced, processed, and cooked meat and seafood products by various researchers [10,11,12,13,14,15,16]. However, to the best of our knowledge, no report is available on the use of BEO as preservatives in meat/meat products. Further, a scanty study has been done on their biological attributes, such as anti-oxidative and antimicrobial properties.

Bamboo, a member of the grass family Poaceae (or Gramineae) has been closely linked to the mankind and civilization since ancient times. The bamboo is utilized as food because they are high in nutrients (proteins, vitamins, carbohydrates, and minerals), yet low in fats and cholesterol. Due to their anti-inflammatory, antioxidative, antibacterial, anti-carcinogenic, cardio defensive, anti-allergic, and vasodilating competencies; bioactive substances, such as phenols, and phytosterols, fatty acids and dietary fibers play a significant role in protecting against many enduring and progressive degenerative ailments [17]. Available reports indicate that bamboo-based products, when consumed regularly, may also aid in lowering the risk of a variety of age-related chronic conditions, including diabetes, Alzheimer’s syndrome and Parkinson’s disease [18].

As consumers are becoming more health conscious, there is a growing demand for meat and meat products free of artificial or chemical additives. Various studies have reported that BEO, apart from managing hypercholesterolemia, i.e., lowering cholesterol levels, exhibit antioxidant and antimicrobial properties [19]. In light of this, Bamboo essential oil (BEO ) may be a natural substitute to synthetic antioxidants and antimicrobials. Hence, the present study was conducted to determine the anti-oxidative and antibacterial potency of BEO utilizing different in vitro systems and studying its in situ effectiveness, based on the physicochemical, microbiological, and sensory characteristics in enhancing the quality, durability and shelf-life of cooked meatballs, stored for 20 days at refrigeration temperature (4 ± 1 °C).

## 2. Materials and Methods

### 2.1. Materials

Fresh boneless chicken meat was obtained from West Bengal Livestock Development Corporation, a Government of West Bengal undertaking company. All the chemicals and reagents used were of analytical grade and purchased from Sigma Aldrich, USA or HiMedia Laboratories, Mumbai, India. Condiments and spice mix were purchased from the local market. Fresh bamboo leaves were collected, dried and ground into fine powder and the essential oil was extracted by a continuous steam distillation of 3 h using a Clevenger-type apparatus. The oil was then dried with anhydrous sodium sulfate and stored in airtight vials at 4 °C until further analysis.

*Bacterial strains*: The test organisms *Bacillus subtilis 3610, Escherichia coli 40*, *Staphylococcus aureus 6571, Vibrio cholerae M010 (0139), Salmonella* Typhimurium *P-4200, Shigella flexneri 29508, Klebsiella pneumoniae 418* and *Proteus vulgaris 6380* received from Microbial Type Culture Collection (MTCC), CSIR-Institute of Microbial Technology, Chandigarh, India were used in this study.

### 2.2. Analysis of Bamboo Essential Oil

#### 2.2.1. Total Phenolics Content

The Folin–Ciocalteu (F-C) technique was used to determine the total phenolics content of BEO with little modification [20]. Briefly, 2.5 mL solution of Folin–Ciocalteu reagent (0.2 N) was mixed with 0.5 mL of BEO at various concentrations dissolved in methanol. After adding 2 mL of sodium carbonate (Na_2_CO_3_) and allowing the solution mixture to stand in the dark for 2 h, the blue color formed was measured spectrophotometrically in terms of the optical density at 760 nm in comparison to a blank. The gallic acid calibration curve was used as standard polyphenol to calculate the total phenolics content of the BEO sample, which was then reported as gallic acid equivalents (GAE), expressed in µg/mL of the sample.

#### 2.2.2. Total Flavonoid Content

The total flavonoid content was determined using a modified version of the aluminium chloride (AlCl_3_) colorimetric method [21]. Briefly, 75 µL of 5% NaNO_2_ solution was mixed with 125 µL of BEO and the mixture was left as such for 6 min. Thereafter, 150 µL of AlCl_3_ (10%) was added and the mixture was incubated for 5 min. To this, 750 µL of (1 M) NaOH was added, and the volume was made up to 2500 µL. The mixture developed pink coloration after 15 min of incubation, and the absorbance at 510 nm was measured. The total flavonoid content was displayed as mg of quercetin equivalents (QE) per mL of the BEO sample.

#### 2.2.3. Fatty Acid Analysis of BEO

The fatty acid composition of BEO was determined by the standard method of AOAC [22] and the values obtained were recorded in percentage for individual fatty acid. 

### 2.3. In Vitro Antioxidant Potential of BEO

#### 2.3.1. DPPH (2,2-diphenyl-2-picrylhydrazyl) Radical Scavenging Activity

The method of Rahman et al. [23] was slightly modified to measure the free radical scavenging activity of BEO against stable 2,2,-diphenyl-2-picrylhydrazyl (DPPH). A well-mixed aliquot of 1 mL sample (diluted with methanol to various concentrations) was added to test tubes along with 2 mL of 0.1 mM DPPH methanolic solution. The change in color (from deep violet to light yellow) indicative of the scavenging activity was then measured spectrophotometrically at 517 nm after it had been incubated in the dark for 30 min. The blank absorbance value was also calculated using methanol. Trolox, BHT, and BHA were employed as positive control. The following equation was used to determine the percentage of inhibition or DPPH scavenging activity:

% Inhibition (I %) = {(A_0_ − A)/A_0_} × 100 (1)

where A_0_ is the blank absorbance and A is absorbance of the test material. The IC_50_ values were calculated from the inhibition percentage (I %) of radical formation in presence of the test substance.

#### 2.3.2. Hydrogen Peroxide (H_2_O_2_) Scavenging Activity

The ability of BEO to scavenge H_2_O_2_ was assessed using a significantly modified version [24]. BEO was diluted with methanol to different concentrations, and 0.1 mL of that sample was transferred to an Eppendorf tube. The volume was then increased to 0.4 mL with 50 mM phosphate buffer (pH 7.4), and 0.6 mL of H_2_O_2_(2 mM) solution was then added. After vortexing, the reaction mixture and incubation for 10 min, the absorbance at 230 nm was measured spectrophotometrically. The BHA, BHT and Trolox served as positive control while methanol served as blank. The percentage of inhibition or H_2_O_2_scavenging activity was calculated, and results were expressed as IC_50_ values.

#### 2.3.3. Ferrous Ion Chelating Activity

The ferrous ion-ferrozine complex method [25] was used to assess the metal ion chelating activity of BEO for ferrous ions (Fe^2+^). A total of 1.5 mL of water and 2 mL of the test sample in methanol (different concentrations) were combined with 25 µL of FeCl_2_ solution (2 mM). After 30 s, the reaction was triggered by adding 50 µL of ferrozine solution (5 mM). Following a thorough shaking, the mixture was incubated at RT for 10 min. At 562 nm, absorbance was measured spectrophotometrically. Trolox was employed as a positive control along with BHT and BHA and methanol as blank. The following formula was used to determine the metal chelating activity: Metal ion chelating rate

(M %) = {1 − (A/A_0_)} × 100, (2)

where A = Absorbance of the Test at 562 nm and A_0_ = Absorbance of the Control at 562 nm. The IC_50_ values were calculated from M %.

#### 2.3.4. Nitric Oxide Radical Scavenging Capacity

The method described by Makhija et al. [26] was adopted. A sodium nitroprusside solution was used to produce nitric oxide radicals by mixing it (1 mL in 10 mM phosphate buffer) with 1 mL of BEO in methanol of various concentrations. The mixture was incubated for 150 min at 25 °C. A total of 1 mL of Griess’ reagent was added to 1 mL of the incubated solution. At 546 nm, absorbance was measured. The oil’s percentage suppression of the nitric oxide radical was calculated with the previously mentioned I % formula and IC_50_ values were presented. BHA, Trolox and BHT were used as positive control and methanol as blank. The mean values of each measurement were computed after being performed in triplicate.

#### 2.3.5. Hydroxyl Radical Scavenging Assay

The hydroxyl radical scavenging capacity of BEO was assessed using the Fenton reaction [27]. In a nutshell, 1 mL of various concentrations of BEO in methanol was combined with 2 mL of 1.5 nM FeSO4, 1 mL of 6 mM hydrogen peroxide, and 0.3 mL of sodium salicylate (20 mM). For 1 h, the reaction mixture was heated to 370 °C. At 510 nm, the reaction mixture’s absorbance was gauged after cooling to RT. As a positive control, Trolox, BHA, and BHT were utilized. IC_50_ values were calculated from I % with the method as mentioned previously.

#### 2.3.6. Superoxide Anion Radical Scavenging

In this test, the reaction mixture contained 0.2 mL of essential oil in methanol having different concentrations, 0.2 mL of 60 mM phenazine methosulfate (PMS), 0.2 mL of 677 mM NADH, and 0.2 mL of 144 mM nitroblue tetrazolium (NBT), all in phosphate buffer (0.1 mol L^−1^, pH 7.4). The absorbance of the reaction mixture was measured at 560 nm. Trolox, BHA, and BHT were used as positive control [28]. Results were expressed as IC_50_ values from I %.

#### 2.3.7. Total Antioxidant Activity by β-Carotene–Linoleic Acid Method

In this assay, antioxidant capacity is determined by measuring the inhibition of the volatile organic compounds and the conjugated diene hydroperoxides arising from linoleic acid oxidation [29]. A stock solution of β-carotene–linoleic acid mixture was prepared as follows: 0.5 mg β-carotene was dissolved in 1 mL of chloroform, 25 µL linoleic acid and 200 mg Tween 40. Chloroform was completely evaporated using a vacuum evaporator. Then, 100 mL of distilled water was added with vigorous shaking; 2.5 mL of this reaction mixture was dispersed to test tubes and 0.5 mL of various concentrations of BEO in methanol were added and the emulsion system was incubated for up to 2 h at 50 °C. The same procedure was repeated with the positive control- BHT, BHA, Trolox and a methanol blank. After this incubation period, the absorbance of the mixtures was measured at 490 nm at an interval of 15 min during the 2 h incubation period.

The rate of β-carotene bleaching for the oil and BHT was calculated according to first-order kinetics, as described in Al-Saikhan et al. [30].

R = ln (1/t × A_t=0_/A_t=t_) (3)

where t is the time in min, A_t=0_ is the initial absorbance of the emulsion immediately after sample preparation (t = 0 min) and A_t=t_ is the absorbance recorded every 15 min after the beginning of the experiment. The average rate of β-carotene degradation (R) was calculated according to first-order kinetics. Results were expressed as IC_50_ values from bleaching or degradation %.

### 2.4. Assessment of In-Vitro Antimicrobial Activity of BEO

#### 2.4.1. Preliminary Antimicrobial Screening of BEO against Microbial Pathogens In Vitro

The BEO was evaluated for its *in-vitro* antimicrobial activity as per Sfeir et al. [31] against eight pathogens and spoilage organisms by Kirby-Bauer disc diffusion method on Mueller-Hinton agar (MHA) plate. Discs were prepared by incorporating 20 µL of BEO of different concentrations (2.5%, 5%, 7.5% and 10%) into each sterile disc (HiMedia, Mumbai, India) separately. Each test organism’s inoculum was prepared in sterile MHA and the turbidity of each suspension was adjusted equal to 0.5 MacFarland standard, which is equal to 1.5 × 10^8^ colony forming units (CFU/mL) for bacteria. Each bacterial culture (20 µL) used in this study was spread on separate MHA plates and the discs containing the above four concentrations of BEO were placed on each MHA plate. The plates were incubated at 37 °C for 18–20 h and the zone of inhibition (ZOI) were measured (in cm) by measuring the diameters of clear zones around the discs. Streptomycin filter discs were used as a control in the study and the ZOI of the BEO was compared with that of the control drug against each bacterium. Each test was performed in triplicate, and the results are expressed in cm ± standard deviation (SD).

#### 2.4.2. In Vitro Minimum Inhibitory Concentration (MIC) and Minimum Biocidal Concentration (MBC)

MIC was determined by the resazurin-based micro-broth dilution method, while MBC was performed following the standard spread plate inoculation method [32,33]. The inocula of each test bacteria were prepared in tryptic soy broth (TSB), following the Clinical and Laboratory Standards Institute (CLSI) guidelines (https://clsi.org/ (accessed on 1 December 2021)), where the OD_600_ value (0.08–0.12) was adjusted, resulting in ~1 × 10^8^ CFU/mL. Then, adjusted inocula were further diluted by 1:100 in TSB, resulting in ~1 × 10^6^ CFU/mL. The initial stock solution of BEO was prepared in methanol at different concentrations. From tubes that had not presented visible turbidity, 10 µL of bacterial inoculum was taken aseptically and was plated. The lowest concentration of BEO which did not produce isolated colonies of the test organisms on inoculated agar plates was considered as the MBC, while the lowest concentration of BEO in broth which changed the colour of Resazurin dye to pink was noted as the MIC. The results are recorded in μL. To determine the nature of the antibacterial effect, the MBC/MIC ratio or tolerance level of the BEO against the different bacterial strains was calculated by MBC/MIC value for the respective bacteria. The tolerance level corresponds to whether the essential oil acts as bacteriostatic or bactericidal for the chosen pathogen. When the ratio was lower than four, the BEO was considered as bactericidal (i.e., kills pathogenic bacteria) and when the ratio was higher than four, it was considered as bacteriostatic (i.e., suppress growth of pathogens).

### 2.5. Assessment of In Situ Food Preservative Effect of BEO in Modified Meat System

#### 2.5.1. Preparation of Cooked Meatballs

Cooked meatballs were prepared as per the standard procedure [5]. Initially, minced meat was mixed properly with sodium chloride and sodium nitrite. After that, all other ingredients such as ice flakes, continents, dry spice mixed, refined wheat flower, salt and essential oils were mixed in a bowl chopper in order to prepare emulsion for meatballs. BEO was added to the meatball emulsions at a concentration of 15 ppm (BEO-I) and 30 ppm (BEO-II). The emulsion without essential oil was considered as control. The levels of all the ingredients used for emulsion preparation are listed in the Supplementary File S1. The meat emulsion from each batch was taken for preparation of meatball (20 g) and boiled in steam until an internal temperature of 75 °C. After boiling, meatballs were cooled for 45 min and stored at 4 °C after packing in polyethylene pouches for evaluation of oxidative, sensory and microbial quality. The processing and preparation of meatballs were repeated three times for each batch (control, BEO-I and BEO-II).

#### 2.5.2. Analysis of Meat Samples

##### pH

The pH of the meatball samples was determined by a combined electrode digital pH meter (Model CP 901, Century Instruments Ltd., India). Approximately 10 gm of meat sample was homogenized with the help of a homogenizer (Omni, Germany) for approximately 1 min in 50 mL of distilled water. The homogenized sample was kept for 5 min and then mixed again by shaking. pH was recorded by immersing the electrode directly into the meat suspension.

##### Proximate Composition and Cooking Yield

The proximate composition such as moisture, protein, ash, and fat contents of control and treated meatballs was analyzed using the standard procedure as per AOAC [34]. The cooking yield was determined as a percentage of initial weight, where 25 g meat emulsion in polyethylene pouch was boiled for 20 min at 80 °C by using a temperature-controlled water bath. After cooking, the emulsion (cooked) was drained of excess water and weight was taken after proper cooking of the meatballs.

##### Color Measurement

Meat samples were cut into slices and immediately the color measurement was carried out on three different spots of slices. The CIE color coordinates such as lightness (L*), redness (a*) and yellowness (b*) were evaluated using MINOLTA Chroma Meter CR-400 (Minolta Co., Ltd., Osaka, Japan).

##### Instrumental Texture Analysis of Cooked Chicken Meatballs

The textural quality measurements of meatballs were done by using an instrumental texture analyzer (Stable Micro System, Model TA HDi, Surrey, UK). Five pieces from the central core of each sample (2 cm × 2 cm × 2 cm) were compressed twice to 80% of the height by a compression probe (P75). A crosshead speed of 2 mm/s was used for the same. The following textural parameters of meatballs were evaluated instrumentally: hardness (N/cm^2^), springiness (cm), cohesiveness (ratio), gumminess (N/cm^2^) and chewiness (N/cm).

##### Expressible Water

The expressible water of meatballs was determined by the method of Jauregui et al. [35] with little modification. Approximately 5 g of meatball sample was placed on two layers of Whatman no. 1 filter paper. The sample was placed in a 50mL centrifuge tube and centrifuged for 15 min at 1500× *g* using a centrifuge (Remi R24, India). Soon after centrifuging, the sample was reweighed and the % of expressible water was calculated by the formula:

Expressible water (%) = Initial weight (g) − Final weight (g)/Initial weight (g) × 100(4)

The samples were analyzed in duplicates and the average value was noted for each sample.

##### Thiobarbituric Acid Reactive Substances

The lipid oxidative stability test (thiobarbituric acid reactive substances-TBARS) was performed during storage according to the standard method of Witte et al. [36] with slight modification. Meat samples (10 g) were homogenized with 25 mL of trichloroacetic acid (TCA) (20%). The homogenized content was filtered with filter paper to get TCA filtrate. An appropriate quantity of filtrate (1.3 mL) was mixed with 3 mL of TBA (5 mM) reagent and boiled for 35 min in a water bath at 70 °C. after cooling under running tap water, the absorbance was taken using a UV-VIS spectrophotometer. The calculation of TBARS value was calculated using a factor of 5.2 and expressed as milligrams of malondialdehyde per kilogram of the sample.

##### Microbiological Analysis

The microbial quality of chicken meatballs was evaluated during storage as per the standard method [37]. The plate count agar was used for enumeration of total viable count (TVC) and media was prepared and sterilized as per the manufacturer’s instructions. Plates after proper incubation showing less than 300 colonies were considered for counting. Microbial count was expressed in log10 cfu/g after multiplying the number of colonies with reciprocal dilution.

##### Sensory Evaluation of Cooked Chicken Meatballs

Sensory evaluation was carried out where 10 trained panelists evaluated the meatballs for various sensory attributes, such as appearance, flavor, juiciness and overall acceptability using an 8-point hedonic scale [38], where 8 = extremely desirable and 1 = extremely undesirable. The meatballs were pre-warmed in a microwave oven for 20 s before sensory evaluation of coded samples at room temperature in separate booths and plain potable water was provided for mouth rinsing in between the samples. 

### 2.6. Statistical Analysis

The measurements of all parameters were done in duplicate, and the study was replicated thrice. The data generated were compiled and analyzed using SPSS Software (Version 20, IBM, USA). The data were subjected to one-way ANOVA for quality parameters and two-way ANOVA for storage parameters. Duncan’s multiple range test was used to compare the means between the treatments, storage period and their interactions for various parameters.

## 3. Results and Discussion

### 3.1. Total Phenolics, Flavonoids and Fatty Acids Content of BEO

Phenolic compounds, the secondary plant metabolites have attracted the interest of the scientific community because of their major antioxidant applications. The major groups of plant phenols include flavonoids, tannins, chalcones, coumarins, phenolic acids etc. Plant phenols signify the largest natural antioxidants mainly because of the various strong hydrogen ion-donating properties of their hydroxyl groups [18,39]. Bamboo are considered a rich source of natural antioxidants. The polyphenols as well as the flavonoids present in bamboo exhibit antioxidant activities via different mechanisms, viz., scavenging of free radicals, quenching of ROS, inhibition of oxidative enzymes, chelation of transition metals etc. [18,40]. Furthermore, the antioxidant capacity of essential oils is related to their phenolic compounds, such as phenolic acids, flavonoids etc. [41]. It is observed that the antioxidant activity of an EO is dependent upon the plant and/or parts used to produce the oil [42]. The antioxidant activities of BEO can be directly correlated to its total phenolics content as reported by researchers for other essential oils [43,44]. The total phenolics and flavonoid contents are the two key indicators widely employed to represent the overall antioxidant activity in the samples. Hence, to shed light, on the bioactive phytochemicals present in the BEO, we evaluated the polyphenols as well as flavonoid content.

Perusal of Table 1 revealed that the total phenolics content was 1244 µg (0.125%/mL of oil) while the flavonoid content was 160.53 µg (0.016%/mL of oil). Similar results were found by Yang et al. [45] in bamboo paste, where the total phenolics content was 23.50 mg/g of bamboo essential oil. Flavonoid content in BEO was found to be much lower than other parts of bamboo plant as evident from other studies [46], where the rhizome sheath showed high content (approximately 10%) while the shoot itself had the least amount (approximately 3%).

Various studies have quantified the total phenolics content in bamboo parts, such as leaves, shoots etc., and some of them have also identified different phenolic compounds from bamboo shoots, such as p-hydrocatechuic acid, catechins, caffeic acid, syringic acid, chlorogenic acid etc., that contribute to the antioxidant activities [19,43,44,46] [19,43,44,46]. In this study, the major fatty acids of BEO were C11:0 (21.82%), C13:0 (18.80%) and C10:0 (16.15%). The fatty acids present in minor quantities in BEO were C6:0, C8:0, C12:0, C14:0, C15:0, C17:0 and C18:1. No PUFAs were found in the oil. Lu et al. [47] also studied the fatty acid composition of bamboo shoot oil and found similar results. Short-chain fatty acids represented the lowest fraction (approx. 7%) followed by long-chain fatty acids (approximately 25%). The medium chain constitutes the highest fraction (nearly 44%) of the total fatty acids. Although one type of monounsaturated fatty acid (MUFA) was predominant, it was in very minimal amounts.

### 3.2. In Vitro Antioxidant Activity of BEO

#### 3.2.1. DPPH Radical Scavenging Activity

DPPH is a stable, synthetic radical that does not disintegrate in water, methanol, or ethanol. The free radical scavenging activities of extracts depend on the ability of antioxidant compounds to lose hydrogen and the structural conformation of these components. The DPPH scavenging activity of BEO was found to be highly potent. The oil scavenged 50% of free radicals at a very low (1.4 µL/mL) concentration (Table 2). The standard antioxidants used were all less effective than the oil. BEO was found to be approximately 10 times more active than BHT and 5 times more than Trolox and BHA, respectively. Likewise, Wang et al. [48] and Singh et al. [42] reported the DPPH scavenging activity (%) of rosemary and cinnamon essential oils to be 62.45% and 78.3%, respectively.

#### 3.2.2. Hydrogen Peroxide (H_2_O_2_) Scavenging Activity

H_2_O_2_ generally undergoes auto-degradation and hence this reagent needs to be freshly and very efficiently prepared with storage in dark amber bottles for antioxidant assay. The efficiency of BEO in scavenging this radical was very high showing 50% inhibition at 2 µL/mL (Table 2). All the control antioxidants chosen were found to be much less potent than the oil. BHT, BHA and Trolox were found to be 43, 25 and 30 times less efficient than oil. So, this essential oil can be used for the prevention of oxidized products in biological systems.

#### 3.2.3. Ferrous Ion Chelating Activity

Reactive oxygen species (ROS) are formed as necessary intermediates of metal-catalyzed oxidation reactions. The transition metal ion Fe^2+^ possesses the ability to perpetuate the formation of free radicals by the gain or loss of electrons. Therefore, the reduction of the formation of reactive oxygen species can be achieved by the chelation of metal ions with chelating agents. Chelation power assays are hence widely carried out to assess the chelation capacity. In our study, BEO showed a 50% reduction in chelation activity at a low concentration of 0.53 µL/mL. BHA showed the same inhibition at approximately 40 µL/mL, BHT and Trolox at 53 and 46.59 µL/mL, respectively (Table 2). Therefore, oil is far more efficacious in the chelation of metals.

#### 3.2.4. Nitric Oxide Radical Scavenging Activity

Numerous physiological and biochemical processes in the human body may produce oxygen-containing free radicals and other reactive oxygen or nitrogen species as by-products. Mainly oxygen species are produced, however, nitrogenous radicals are also generated sometimes producing oxidative stress. Analogous to previous results, this also showed that BEO can scavenge half of the generated nitrite radicals at very low concentration (1.615 µL/mL). While the standard antioxidants employed showed almost 50 times less efficacy in scavenging the free radicals (i.e., 43 to 49). So, the control antioxidants showed indifferent or almost similar efficiency in scavenging nitrite anion or similar such radicals in vitro.

#### 3.2.5. Hydroxyl Radical Scavenging Assay

Hydroxyl radical (^∙^OH), is now known to be the most biologically active free radical and is formed in vivo under hypoxic conditions. This method measured the ability of the oil to scavenge the most versatile free radical generated in the cellular environment. BEO scavenged 50% radicals at 1.23 µL/mL while BHT, BHA and Trolox inhibited similar amounts at much high doses of 82.19, 51.36 and 69.14 µL/mL, respectively (Table 2).

#### 3.2.6. Superoxide Anion Radical Scavenging

The production of superoxide anion radical (O_2_•^−^) is essential for the life of aerobic organisms. This free radical acts as a signaling molecule, regulating numerous biological processes, including apoptosis, ageing, and senescence. Nevertheless, when overproduction of O_2_•^−^ occurs and/or antioxidant defenses are deficient, oxidative stress may develop, damaging important biomolecules and altering their physiological function. These effects have been associated with the development of several diseases. The BEO was very potent as shown by its capacity to scavenge such superoxide radicals (50%) at 1.232 µL/mL (Table 2). While BHT showed 50 times lower scavenging activity, BHA and Trolox showed 32 and 38-times lower activity, respectively.

#### 3.2.7. Total Antioxidant Activity by *β*-Carotene–Linoleic Acid Method

The bleaching test of β-carotene is based on the loss of β-carotene’s yellow color due to its reaction to radicals generated by acid oxidation linoleic as an example in an emulsion. The presence of an antioxidant could neutralize free radicals derived from acid linoleic acid and therefore prevent the oxidation and bleaching of β-carotene. BEO was found to be approximately 81, 54 and 63 times more efficient in prevention of oil than BHT, BHA and Trolox, respectively.

### 3.3. Antimicrobial Activity of BEO by Disc Diffusion Method

**Figure 1:**
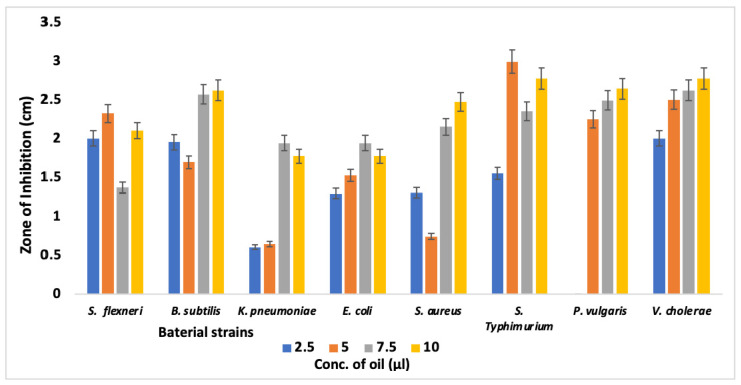
Zone of inhibition of BEO by disc diffusion method against different pathogenic bacterial strains. Values are expressed as mean ± SEM (n = 3). Eight bacterial pathogens were screened for their clear zone in plates treated with four different concentrations of essential oil. Large zones of inhibition indicate that the organism is susceptible, while a small or no zone of inhibition indicates resistance.

To gain insights into the preliminary antibacterial activity of BEO against pure pathogenic bacterial strains, we utilized disc diffusion methods on agar plates. For ease in handling, the four concentrations of BEO (2.5%, 5%, 7.5% and 10%) were checked for preliminary antimicrobial activity against all the bacterial strains. *S. flexneri* showed an increased inhibition zone with increased concentration (Figure 1). In *K. pneumoniae,* lower concentration of 2.5 and 5 µL showed almost similar zones of inhibition, while that at 7.5 µL increased approximately 3.5-fold and at 10 µL approximately 3-fold higher. The highest concentration showed a lower diameter than the penultimate concentration. The highest concentration (10 µL) of BEO in *E. coli* showed the same zone of diameter as that of *K. pneumoniae*. With increasing concentration of BEO in *E. coli*, inhibition gradually increased but at 10 µL, it decreased. *S. aureus* showed inhibition in all concentrations with minimal at 5 µL and maximum at 10 µL. *S. Typhimurium* was found to be the most susceptible of all the bacterium. The 5 µL concentration showed the highest zone of inhibition among all, while 2.5 µL showed half zone of inhibition to that. *P. vulgaris* at 2.5 µL showed no inhibition, but with a gradual increase in concentration, the zone of inhibition gradually increased, but there was not much difference between the diameters. The *V. cholerae* showed an increase in inhibition with increasing concentration of BEO. So, Gram-positive as well as Gram-negative bacteria were efficiently inhibited by BEO, with Gram-negative pathogens more susceptible to inhibition as compared to Gram-positive bacteria.

The 10% BEO had the largest ZOI (2.63 cm) against *B. subtilis*, followed by 2.48 cm against *S. aureus*, 1.78 cm and 1.78 cm against *K. pneumoniae* and *E. coli*, respectively. The activity of BEO against most bacteria was found to be varying in a concentration-dependent manner (Figure 1). Hence, it can be concluded that BEO exhibited a broad-spectrum antimicrobial activity against both Gram-positive and Gram-negative food-borne bacteria and may be explored as a promising natural antimicrobial in the food sector. Similar results were also observed by Tao et al. [49,50]. Tanaka et al. [51] reported that the active constituents in bamboo extract that inhibited the growth of *S. aureus* and *E. coli* were stigmasterol and dihydro-brassicasterol. Similarly, Tao et al. [49] concluded that the antimicrobial activity of bamboo essential oil may be due to the disruption of membrane integrity of food-borne bacteria, such as *B. subtilis*, *S. aureus* and *E. coli*. Although the exact mechanism of action for the antibacterial activity of BEO is not entirely clear, membrane disruption by phenolics and metal chelation by flavonoids might have contributed to the antimicrobial activity of BEO in the current study.

### 3.4. Determination of MIC and MBC of BEO

The MIC values of BEO were much less and found to 1.5 µL for all the tested bacteria used in this study. The MIC values of BEO against *B. subtilis*, *E. coli*, *P. vulgaris*, *Shigella flexneri*, *K. pneumoniae*, *V. cholerae* and *S. aureus* are given in Table 3. As it is known that MIC is a measure of the antimicrobial performance [50,52,53], so smaller MIC corresponds to potent and efficient antimicrobial effect. So, the lower concentrations of BEO for killing or mortality of bacteria shows that the oil can stop growth and reproduction at very minimal doses. Possas et al. [54] and Burt [55] found that higher concentrations of essential oils are required to achieve the same antimicrobial effect in food as *in-vitro*, due to the interaction of hydrophobic essential oils with various food components. Keeping this in mind and considering the acceptable sensory scores of preliminary trials, BEO was incorporated at 15 ppm (BEO-I) and 30 ppm (BEO-II) in cooked chicken meatballs in the current study.

### 3.5. Incorporation of BEO in Chicken Meatballs

#### 3.5.1. Effect of BEO on pH, Emulsion Stability, Cooking Yield and Proximate Composition

Bamboo parts have been used for the bio-preservation of meat and meat products by several researchers [56,57,58,59]. Nowadays, essential oils are explored for their antioxidant, antimicrobial and beneficial human health effects and are being incorporated into functional meat and meat products [41,53,60,61,62]. The BEO, derived from the different part of bamboo has also been tested in vitro for its antimicrobial, antioxidant and other health-promoting effects [47,49,50]. However, no study has been done to evaluate the effect of BEO on the quality characteristics of meat and meat products, especially during refrigerated storage.

Considering the above facts, this study was planned to evaluate the effect of BEO in cooked chicken meatballs under refrigerated storage. Based on the results of preliminary trials conducted in the laboratory, the levels of incorporation of BEO were optimized by conducting a series of sensory evaluations, physico-chemical analysis, antimicrobial and antioxidant assays. Finally, BEO was incorporated at 15 ppm (BEO-I) and 30 ppm (BEO-II) in chicken meatballs and its effect was studied on the oxidative and microbial stability of meatballs during refrigerated storage (4 ± 1 °C) for 20 days as compared to the control.

The data presented in Table 4 suggest that there was no significant difference (*p* > 0.05) in control and BEO-treated products in terms of emulsion pH, emulsion stability, cooking yield, proximate composition (moisture, protein, fat and ash) and expressible water. The emulsion pH was 6.10 ± 0.02, 6.09 ± 0.02 and 6.11 ± 0.01 for control, BEO-I and BEO-II treated products, respectively. The emulsion stability (%) values were 95.08 ± 0.12, 95.12 ± 0.14 and 95.02 ± 0.11 for control, BEO-I and BEO-II treated products, respectively. There was a non-significant (*p* > 0.05) increase in the cooking yield (%) of the treatments than control and the values were 96.79 ± 0.07, 97.26 ± 0.09 and 97.83 ± 0.22 for control and treatments BEO-I and BEO-II, respectively. The slightly higher cooking yield in the treated samples might be because of better water retention capacity due to addition of BEO to the meat balls. The moisture, protein, fat and ash contents of the control and treatments were in the range of 67.74–68.26%, 15.88–16.04%, 13.76–14.69% and 1.08–1.12%, respectively, and the values showed non-significant (*p* > 0.05) differences.

The expressible water percentages (%) were 26.84, 25.72 and 26.96 for control, BEO-I and BEO-II treated products, respectively. There were non-significant (*p* > 0.05) differences in expressible water % of the control and treated products. The moisture content and expressible water % followed similar behavior because these variables are intrinsically linked [63]. The reason behind non-significant differences among the control and treated products for the above parameters might be the very little amount of BEO used to alter the physico-chemical characteristics of the meat emulsion. These findings are in complete agreement with the results of various workers who worked on essential oils and the quality of meat products [12,64,65].

On the other hand, the total phenolics contents of the control, BEO-I and BEO-II were 0.074, 1.484 and 1.852 mgGAE/g, respectively. A significant difference (*p* < 0.05) was observed in the total phenolics content of the control and BEO-treated products. The reason might be due to the high total phenolics content of BEO [47,50]. Sharma et al. [66] also reported that the clove EO and cassia EO incorporated in fresh chicken sausages had significantly higher total phenolic contents than the control. Similar results were also reported by many researchers who used different natural antioxidants in meat and meat products [15,38,67].

#### 3.5.2. Effect of BEO on Textural Properties of Meatballs

From the data presented in Table 4, it is observed that the incorporation of BEO at 30 ppm (BEO-II) significantly (*p* < 0.01) improved the textural properties of chicken meatballs as compared to BEO-I and the control. Significantly lower values of springiness, cohesiveness, gumminess and chewiness were observed in BEO-II products as compared to BEO-I and control. The springiness and cohesiveness values of BEO-I were in between the values for control and BEO-II. Additionally, there was a non-significant reduction in hardness values of BEO-treated products as compared to the control and the value ranged from 42.75 to 38.97. The improvement in textural properties of chicken meatballs may be due to the addition of oil to the product that reduced the strength of protein-gel mixture. The results were in complete agreement with the findings of Thomas et al. [68] and Kim et al. [69] in their experiments on the effect of fermented bamboo shoot mince on the quality of pork nuggets and the effect of soy sauce on quality of pork patties, respectively. Tomović et al. [70] also observed improved instrumental textural qualities of dry fermented sausage upon the addition of essential oil.

### 3.6. Effect of BEO on Physico-Chemical and Microbiological Qualities of Chicken Meatballs

#### 3.6.1. Effect of BEO on pH of Meatballs

There was a gradual and significant (*p* < 0.05) increase in the pH of the control and treated products with the advent of storage days. The pH value ranged from 6.14–6.58, 6.14–6.29 and 6.15–6.31 for control, BEO-I and BEO-II from day 0 to day 20 of refrigerated storage (Table 5). The pH values of treated products were still under the acceptable level, whereas that of the control product just exceeded the higher limit of acceptance at the end of the storage period. The rate of pH increase was highest for the control, which may be due to the formation of amino compounds during the proteolysis caused by the growth of microorganisms [70]. The pH increase in all the test groups with the advancing storage days may be due to the fact that, as the stored glucose gets completely depleted, the microbes utilize amino acids released by protein degradation. The ammonia and other amino group compounds released as amino acid degradation products lead to an increase in the pH of the treated meatballs. The rate of pH change was significantly (*p* < 0.05) lower in BEO-I treated product, and BEO-II product exhibited the lowest rate of pH change throughout the storage period. This may be due to the antimicrobial and antioxidant activities of BEO [12]. Similar results in pH change after the incorporation of bamboo parts in meat and meat products were also observed during storage by several researchers [59,64,71,72].

#### 3.6.2. Effect of BEO on TBARS Value of Meatballs 

Perusal of Table 5 revealed that the mean TBARS values ranged from 0.34–1.67, 0.34–0.73 and 0.34–0.64 mg malondialdehyde/kg for control, BEO-I and BEO-II, respectively, from 0 day to 20 day of storage period. On day 0 of storage, there was a non-significant difference (*p* > 0.05) among the TBARS values of control and treated samples. However, TBARS values in all the test samples showed a significant (*p* < 0.05) and gradual increasing trend, as the storage days progressed. These results suggested that though the rate of lipid oxidation was retarded in the treated meat balls, it could not be completely inhibited and showed a gradual increasing pattern even at the refrigerated storage. This could be due to the increase in lipid oxidation (non-microbial origin) and indirectly due to microbial growth (evident from the values of total viable count as given in Table 5) with advancing storage period [67].

The rate of increase in TBARS value was significantly (*p* < 0.05) highest in control as compared to test products and BEO-II (T2) had significantly (*p* < 0.05) the lowest rate of increase in TBARS value during the entire storage period. The control sample had the highest TBARS values which may be due to the unavailability of any antioxidant in it. Further, the mean TBARS values of the treated meatballs were below the standard acceptable level of 1–2 mg malonaldehyde/kg of meat throughout the storage period [71]. This could be attributed to the high antioxidant activities and total phenolics content of BEO used in the treated products that retarded lipid oxidation in meatballs [12,38,67]. Similar to other plant antioxidants, the phenolic and flavonoid compounds present in BEO had a great free radical scavenging, metal ion chelating and singlet oxygen quenching activity (Table 2), thereby lowering the lipid peroxidation and TBARS values of the treated meatball samples as compared to control. These results bear similarity with the findings of Thomas et al. [68] in bamboo shoot extract-treated pork nuggets. In a different study, Sharma et al. [12] also noticed a similar trend in different EO-treated chicken sausages.

#### 3.6.3. Effect of BEO on Color Values of Meatballs

The mean lightness values (L*) of the control, T1 and T2 ranged between 67.63–68.45, 67.41–67.94 and 67.59–67.92, respectively, from day 0 to day 20 of storage (Table 5). There was a non-significant (*p* > 0.05) increase in lightness value (L*) observed in the control as well as treated products with increasing storage periods. However, the treated products displayed slightly lower values of lightness than the control. The increase in lightness value with the progress of the storage period may be due to the breakdown of myoglobin. Pateiro et al. [63] also suggested that the increase in lightness value (L*) may be due to moisture loss from the surface during storage. However, the slightly lower values of lightness values (L*) of treated products may be due to the interaction between the bioactive compounds (phenols, terpenes, flavonoids etc.) present in the BSO and myoglobin [70]. The addition of bamboo shoot extract also decreased the lightness value (L*) of pork nuggets [68]. The mean redness (a*) values ranged between 8.31–7.45, 8.43–8.15 and 8.32–8.14, respectively, for control, T1 and T2. There was a significant (*p* < 0.05) decrease in redness (a*) values of the control and treated products with the advent of storage days, however, the treated samples had significantly (*p* < 0.05) increased redness (a*) values than control. This could be attributed to the increased conversion rate of myoglobin to metmyoglobin in the treated meatballs at a comparatively lower pH than control meatballs [73]. These results are in corroboration of the findings of Tomović et al. [70] in essential oil-incorporated dry fermented sausages. Further, the results are also in accordance with the findings of Pateiro et al. [63] who reported that natural antioxidants are effective in preserving the redness (a*) values of *chorizo*, a Spanish dry-cured sausage.

The mean yellowness (b*) values ranged from 18.42–18.36, 18.50–18.37 and 18.32–18.22 for the control, T1 and T2, respectively from day 0 to day 20 of the storage period (Table 5). There was a non-significant (*p* > 0.05) decreasing trend of yellowness (b*) values observed in the control as well as treated products with increasing storage periods. The results are also in accordance with the findings of various researchers [63,70,73]. The slightly higher yellowness (b*) values of treated products may be attributed to the oil base and antioxidants present in BEO [69].

#### 3.6.4. Effect of BEO on Microbiological Changes in Meatballs

The total viable count (TVC) in the control sample increased from the initial count of 2.67 log cfu/g to 6.39 log cfu/g at the end of the storage study. The mean TVC values of T1 and T2 were in the range of 2.64–4.13 log cfu/g and 2.61–4.07 log cfu/g, respectively, from the beginning to the end of the storage period (Table 5). The initial low values of TVC of both control and treatments might be due to steam cooking of meatballs, good handling practices and hygienic laboratory conditions on the day of processing. The TVC value of control was the highest and that of T2 was the lowest at all storage intervals. The data presented in Table 5 suggest that storage time significantly (*p* < 0.05) affected the TVC values and there was a significant (*p* < 0.05) gradual increase in TVC values of control, T1 and T2. This might be due to an increase in microbial growth with increasing pH values and lipid peroxidation and protein denaturation of cooked chicken meatballs (given in Table 2). The TVC value of the control product showed significantly (*p* < 0.05) higher values than treatments during the entire storage period. The slow increase in TVC and lower values of TVC in T1 and T2 may be attributed to the antimicrobial activities of BEO as also reported by others [47,50,65]. The findings further suggest that the antimicrobial activity of BEO against common foodborne pathogenic and spoilage bacteria (Table 3) could have affected the TVC counts of the test products during the refrigerated storage period. Therefore, BEO treated meatballs had a prolonged refrigerated shelf life as a result of increased lag phase and retarded log phase of viable bacteria.

The values also indicate that the TVC of T1 and T2 on day 20 were below the threshold limits and may be considered as microbiologically safe. However, the TVC value of a control on day 20 was beyond the acceptable limits, hence the product may be considered as spoiled. These results are in complete agreement with the findings of Moraes-Lovison et al. [64] and Siewe et al. [65] in their experiments on different EO incorporation in several meat products. Similar results are also reported by different workers [61,64,68,69,70] in different experiments in meat and meat products.

#### 3.6.5. Effect of BEO on Sensory Attributes of Meatballs

Cooked chicken meatballs incorporated with different levels of BEO were evaluated for different sensory attributes and the results are presented in Table 6. There was a significant (*p* < 0.05) decrease in sensory scores of all sensory attributes for all the test groups with advancing storage days. After 10 days of refrigerated storage, significant (*p* < 0.05) differences in sensory scores of all sensory attributes were observed for control and BEO-treated products. The mean appearance, flavor, juiciness and overall acceptability scores of T1 obtained the highest values during advancing storage days (i.e., from day 5 and onwards), followed by that of T2, whereas the control product obtained the lowest scores for all attributes during the storage period.

The mean appearance scores for control, T1 and T2 ranged from 6.69–5.41, 6.71–6.43 and 6.70–6.37, respectively, from day 0 to day 20 of refrigerated storage. There was a significant (*p* < 0.05) decrease in mean appearance scores of T1 and T2 from 15 days of storage, whereas that of the control product showed a significant (*p* < 0.05) decrease from day 10 onwards. Although significant (*p* < 0.05) differences in appearance scores of all the groups (C, T1 and T2) were observed from day 10 of the storage study, the values for T1 and T2 were comparable. The fact may be due to the protective effect of BEO (antioxidant and antimicrobial) on color and appearance fading of cooked chicken meatballs. The instrumental color values of control and treated products as discussed in Table 5 also bear similarity with these findings. Similar results were also obtained by different workers [12,67,71,72] in their experiments related to the incorporation of different EOs in vacuum-packaged fresh chicken sausages, drumstick flowers in chicken meat nuggets and bamboo shoot paste in beef patties, respectively.

The mean flavor scores for control, T1 and T2 ranged from 7.04–4.94, 7.05–6.44 and 7.06–6.39, respectively, from day 0 to day 20 of refrigerated storage (Table 6). From the day of processing (0 day) to 15th day of storage, the flavor scores of BEO-incorporated chicken meatballs were higher with the higher levels of BEO addition. A significant (*p* < 0.05) decrease in flavor scores for control andT1 was observed since day 10 and that for T2 was evident since day 5 in a gradual manner with the progression of storage days. The individual flavor scores of the control, T1 and T2 decreased significantly (*p* < 0.05) from day 10 of the storage study till day 20. However, on day 20, T1 obtained the highest mean flavor scores, and it was comparable to T2, but the flavor scores of the control product were significantly (*p* < 0.05) lower than the treatment groups. This shows that the flavor imparted due to the addition of BEO to cooked chicken meatballs was very much desirable. The gradual decrease in flavor scores of control and test products may be due to their increasing lipid-protein oxidation and high microbial loads (Table 5) that release unpleasant flavor compounds, such as H_2_S, ammonia, amines, malonaldehyde etc. The significantly higher flavor scores of BEO-treated products may be due to flavor compounds [39,43] present in BEO. Similar flavor scores were also obtained by Thomas et al. [68] and Wan Rosli and Habibah [71] in bamboo shoot-incorporated pork meat nuggets and beef patties, respectively.

The mean juiciness scores of the control, T1 and T2 were in the ranges of 7.01–5.40, 7.03–6.37 and 7.09–6.32, respectively, from day 0 to day 20 of refrigerated storage. The juiciness scores of BEO-incorporated chicken meatballs were higher with the higher levels of BEO addition and were comparable to control on 0 day. However, from day 5, there was a significant (*p* < 0.05) decrease in juiciness scores of controls and treated products; however, T1 obtained highest scores among all samples during the storage intervals. The significantly (*p* < 0.05) lower values in juiciness scores of the control product were evident since day 15 than that of T1 and T2, which were comparable to each other. The significant gradual moisture loss during storage may be the probable reason for the decrease in juiciness of the control as well as treated samples with the progressing storage days. The higher juiciness scores of treated products may be attributed to high moisture content of BEO-treated products (Table 6). Similar results were also obtained by Das et al. [59], Wan Rosli and Habibah [71] in fermented bamboo shoots incorporated spent hen nuggets and beef patties, respectively.

The mean overall acceptability scores of the control, T1 and T2 were in the ranges of 6.89–5.25, 6.95–6.56 and 7.05–6.39, respectively, from day 0 to day 20 of refrigerated storage. The overall acceptability scores showed a similar decreasing trend in appearance, flavor and juiciness with progress in storage days. Significantly (*p* < 0.05) higher overall acceptability scores were noticed in the BEO-treated samples than in the control with progress in storage days which may be due to the combination of higher appearance, flavor and juiciness scores of treated products (Table 6). However, there was a significant (*p* < 0.05) decrease in overall acceptability scores of all the test samples with the advancement of storage days, which may be due to an increase in lipid oxidation, protein degradation and higher microbial load in the products. Similar results were also obtained by Das et al. [59] and Thomas et al. [73] in bamboo shoot incorporated spent hen and pork meat nuggets, respectively. The sensory scores obtained in this study are in conformity with the findings of various researchers [56,57,58,59,68,71,73,74] who incorporated different bamboo parts in different meat and meat products.

The results of storage study suggest BEO incorporated chicken meatballs offered more promising results in terms of physico-chemical, microbiological and sensory evaluation parameters as compared to control products. The values of T1 (meatballs with 15 ppm BEO) and T2 (meatballs with 30 ppm BEO) were almost comparable during the entire storage study and were significantly (*p* < 0.05) higher than the control for almost all the parameters. However, meatballs with 15 ppm BEO (T1) received non-significantly higher sensory scores than meatballs treated with 30 ppm (T2) from day 5 of storage till day 20.

## 4. Conclusions

From the study, it can be concluded that the ability of the essential oil to scavenge different free radicals is noteworthy. The bamboo essential oil used in this study is potent and displayed good antioxidant as well as antibacterial activity. This may be useful in the food and pharmaceutical industries in the treatment of oxidative-related ailments as well as in preservation domains. From this study, it is apparent that BEO can be incorporated into cooked chicken meatballs to improve the physico-chemical, microbiological and sensory attributes of meat products. The study recommends that the addition of a minimum amount of BEO, i.e., 15 ppm in meatballs is optimum in effectively improving the storage stability and quality of cooked chicken meatballs. This study further revealed that cooked chicken meatballs with BEO had a shelf life of 20 days at refrigerated storage (4 ± 1 °C). As natural bioactive components are the need of the hour, BEO can be used to replace synthetic and artificial food preservatives. Therefore, the untapped potential of BEO, a benign and non-toxic preservative may be explored further for the bio-preservation and the development of functional meat and meat products.

## Figures and Tables

**Table 1:** Total phenolics, flavonoid content and fatty acid composition of BEO.

Phytochemicals	Value
Total phenolics (µg GAE/mL of BEO)	1244
Total flavonoid (µgQE/mL of BEO)	160.53
**Fatty Acid**	**Percentage of Total Fatty Acids**
Caproic acid (C6:0)	6.935
Caprylic acid (C8:0)	3.825
Capric acid (C10:0)	16.155
Undecylic acid (C11:0)	21.82
Lauric acid (C12:0)	1.53
Tridecylic acid (C13:0)	18.785
Myristic acid (C14:0)	1.06
Pentadecylic acid (C15:0)	1.225
Margaric acid (C17:0)	1.045
Oleic acid (C18:1)	3.355

GAE = Gallic acid equivalent; QE = Quercetin equivalent.

**Table 2:** Antioxidant assays determined of BEO in terms of scavenging activities of different free radicals.

In Vitro Antioxidant Potential *	BEO	BHT	BHA	Trolox
DPPH scavenging activity	1.393 ± 0.061	13.025 ± 0.244	6.754 ± 0.166	6.86 ± 0.113
H_2_O_2_ scavenging activity	1.999 ± 0.019	85.231 ± 1.136	52.466 ± 0.004	61.137 ± 0.627
Ferrous ion chelating activity	0.536 ± 0.002	52.933 ± 0.047	39.921 ± 0.009	46.595 ± 0.180
Nitric Oxide Radical Scavenging activity	1.615 ± 0.041	78.638 ± 0.552	69.432 ± 0.185	73.645 ± 0.265
Hydroxyl radical scavenging activity	1.792 ± 0.027	82.1945 ± 0.029	51.363 ± 0.297	69.147 ± 0.376
Superoxide anion radical scavenging activity	1.232 ± 0.005	63.702 ± 0.037	39.309 ± 0.028	46.242 ± 0.296
Total antioxidant activity	0.865 ± 0.017	70.113 ± 0.147	46.846 ± 0.384	54.544 ± 0.503

* IC_50_ value in terms of µL/mL or µg/mL (n = 3).

**Table 3:** MIC and MBC values and tolerance of BEO for different bacterial strains.

Bacteria	MIC (µL)	MBC (µL)	Tolerance Level	Inference
*Shigella flexneri*	1.5	6	4	Bactericidal
*Bacillus subtilis*	1.5	7	4.66	Bactericidal
*Klebsiella pneumoniae*	1.5	7	4.66	Bactericidal
*Escherichia coli*	1.5	6	4	Bactericidal
*Staphylococcus aureus*	1.5	6	4	Bactericidal
*Proteus vulgaris*	1.5	8	5.33	Bactericidal
*Vibrio cholerae*	1.5	7	4.66	Bactericidal
*Salmonella* Typhimurium	1.5	5	3.33	Bacteriostatic

**Table 4:** Effect of BEO on emulsion pH, emulsion stability, cooking yield, proximate composition, and texture profile of chicken meatballs.

Parameters	Control	BEO-I (T1)	BEO-II (T2)
***Emulsion Stability, Cooking Yield, Proximate Composition***			
Emulsion pH	6.10 ± 0.02	6.09 ± 0.02	6.11 ± 0.01
Emulsion stability (%)	95.08 ± 0.12	95.12 ± 0.14	95.02 ± 0.11
Cooking yield (%)	96.79 ± 0.07	97.26 ± 0.09	97.83 ± 0.22
Moisture (%)	68.26 ± 0.64	67.86 ± 0.72	67.74 ± 0.68
Protein (%)	15.88 ± 0.44	15.92 ± 0.34	16.04 ± 0.50
Fat (%)	13.76 ± 0.49	14.06 ± 0.35	14.69 ± 0.62
Ash (%)	1.08 ± 0.49	1.12 ± 0.13	1.09 ± 0.89
Total phenolics content (mg GAE/g) **	0.074 ± 0.16 ^b^	1.484 ± 0.12 ^a^	1.852 ± 0.18 ^a^
Expressible water (%)	26.84 ± 1.12	25.72 ± 3.00	26.96 ± 2.05
***Textural Properties of Meatballs***			
Hardness (N/cm^2^)	42.75 ± 1.07	42.45 ± 1.86	38.97 ± 1.89
Springiness (cm)	0.90 ± 0.01 ^a^	0.89 ± 0.02 ^ab^	0.87 ± 0.01 ^b^
Cohesiveness (ratio)	0.65 ± 0.02 ^a^	0.63 ± 0.02 ^ab^	0.61 ± 0.03 ^b^
Gumminess (N/cm^2^)	27.27 ± 0.74 ^a^	27.20 ± 0.97 ^a^	24.86 ± 0.23 ^b^
Chewiness (N/cm)	24.43 ± 0.75 ^a^	23.84 ± 0.82 ^a^	21.72 ± 0.43 ^b^

Control = No BEO; BEO-I (T1) = 15 ppm bamboo essential oil and BEO-II (T2) = 30 ppm bamboo essential oil; n = 6; ** *p* < 0.01; (a–b) mean ± SE with superscript (a–b) on the same row are significantly different (*p* < 0.05).

**Table 5:** Effect of BEO on physico-chemical and microbiological qualities of chicken meatballs.

Treatments	Day 0	Day 5	Day 10	Day 15	Day 20
***pH***					
Control	6.14 ± 0.02 ^e^	6.19 ± 0.01 ^dA^	6.41 ± 0.01 ^cA^	6.49 ± 0.01 ^bA^	6.58 ± 0.01 ^aA^
BEO-I (T1)	6.14 ± 0.01 ^d^	6.16 ± 0.03 ^dB^	6.21 ± 0.01 ^cB^	6.26 ± 0.01 ^bB^	6.29 ± 0.01 ^aB^
BEO-II (T2)	6.15 ± 0.01 ^b^	6.17 ± 0.01 ^abAB^	6.22 ± 0.01 ^aB^	6.27 ± 0.01 ^aB^	6.31 ± 0.01 ^aB^
***Thiobarbituric Acid Reactive Substance (TBARS) Value***					
Control	0.34 ± 0.01 ^e^	0.54 ± 0.01 ^dA^	0.73 ± 0.01 ^cA^	1.15 ± 0.02 ^bA^	1.67 ± 0.03 ^aA^
BEO-I (T1)	0.34 ± 0.01 ^e^	0.38 ± 0.01 ^dB^	0.45 ± 0.01 ^cB^	0.54 ± 0.01 ^bB^	0.73 ± 0.01 ^aB^
BEO-II (T2)	0.34 ± 0.01 ^e^	0.37 ± 0.01 ^dB^	0.42 ± 0.01 ^cB^	0.48 ± 0.01 ^bC^	0.64 ± 0.01 ^aC^
Lightness (L*)					
**Control**	**67.63 ± 0.10**	**67.90 ± 0.07**	**68.08 ± 0.15**	**68.25 ± 0.12 ^A^**	68.45 ± 0.08 ^A^
BEO-I (T1)	67.41 ± 0.33	67.57 ± 0.28	67.67 ± 0.20	67.80 ± 0.12 ^B^	67.94 ± 0.16 ^B^
BEO-II (T2)	67.59 ± 0.15	67.78 ± 0.11	67.81 ± 0.05	67.87 ± 0.14 ^B^	67.92 ± 0.09 ^B^
Redness (a*)					
**Control**	**8.31 ± 0.07 ^a^**	**8.28 ± 0.05 ^ab^**	**8.13 ± 0.04 ^bB^**	**7.96 ± 0.04 ^cB^**	7.45 ± 0.07 ^dB^
BEO-I (T1)	8.43 ± 0.10 ^a^	8.35 ± 0.03 ^a^	8.30 ± 0.04 ^aA^	8.27 ± 0.04 ^abA^	8.15 ± 0.02 ^abA^
BEO-II (T2)	8.32 ± 0.04 ^a^	8.29 ± 0.02 ^ab^	8.24 ± 0.02 ^abA^	8.21 ± 0.04 ^bcA^	8.14 ± 0.03 ^cA^
Yellowness (b*)					
Control	18.42 ± 0.08	18.40 ± 0.07	18.39 ± 0.03	18.37 ± 0.01	18.36 ± 0.04
BEO-I (T1)	18.50 ± 0.09	18.47 ± 0.07	18.44 ± 0.05	18.41 ± 0.04	18.37 ± 0.04
BEO-II (T2)	18.32 ± 0.11	18.30 ± 0.11	18.28 ± 0.10	18.24 ± 0.09	18.22 ± 0.09
***Total Viable Count***					
Control	2.67 ± 0.03 ^e^	3.83 ± 0.06 ^dA^	4.92 ± 0.02 ^cA^	5.79 ± 0.03 ^bA^	6.39 ± 0.02 ^aA^
BEO-I (T1)	2.64 ± 0.04 ^e^	2.87 ± 0.02 ^dB^	3.17 ± 0.03 ^cB^	3.79 ± 0.04 ^bB^	4.13 ± 0.03 ^aB^
BEO-II (T2)	2.61 ± 0.03 ^e^	2.76 ± 0.03 ^dB^	3.04 ± 0.04 ^cC^	3.49 ± 0.03 ^bC^	4.07 ± 0.06 ^aB^

Control = No BEO; BEO-I (T1) = 15 ppm bamboo essential oil and BEO-II (T2) = 30 ppm bamboo essential oil. (a–e) mean ± SE with superscript (a–e) on the same row are significantly different (*p* < 0.05). n = 6. (A–C) mean ± SE with superscript (A–C) on the same column are significantly different (*p* < 0.05).

**Table 6:** Effect of bamboo essential oil (BEO) on sensory qualities of cooked chicken meatballs.

Treatments	Day 0	Day 5	Day 10	Day 15	Day 20
***Appearance***					
Control	6.69 ± 0.04 ^a^	6.66 ± 0.03 ^a^	6.22 ± 0.12 ^bB^	6.06 ± 0.03 ^cB^	5.41 ± 0.05 ^dB^
BEO-I (T1)	6.71 ± 0.04 ^a^	6.64 ± 0.03 ^a^	6.60 ± 0.04 ^aA^	6.48 ± 0.03 ^bA^	6.43 ± 0.02 ^bA^
BEO-II (T2)	6.70 ± 0.03 ^a^	6.62 ± 0.03 ^a^	6.61 ± 0.04 ^aA^	6.50 ± 0.11 ^abA^	6.37 ± 0.06 ^bA^
***Flavor***					
Control	7.04 ± 0.16 ^a^	6.81 ± 0.15 ^a^	6.25 ± 0.07 ^bB^	6.06 ± 0.05 ^bB^	4.94 ± 0.10 ^cB^
BEO-I (T1)	7.05 ± 0.09 ^a^	6.87 ± 0.06 ^a^	6.75 ± 0.13 ^abA^	6.63 ± 0.07 ^bcB^	6.44 ± 0.05 ^cA^
BEO-II (T2)	7.06 ± 0.05 ^a^	6.93 ± 0.05 ^ab^	6.75 ± 0.07 ^bcA^	6.67 ± 0.06 ^cA^	6.39 ± 0.09 ^dA^
***Juiciness***					
Control	7.01 ± 0.07 ^a^	6.62 ± 0.09 ^b^	6.56 ± 0.09 ^b^	6.00 ± 0.07 ^cB^	5.40 ± 0.12 ^dB^
BEO-I (T1)	7.03 ± 0.12 ^a^	6.86 ± 0.11 ^ab^	6.69 ± 0.10 ^bc^	6.56 ± 0.09 ^cdA^	6.37 ± 0.10 ^dA^
BEO-II (T2)	7.09 ± 0.07 ^a^	6.80 ± 0.07 ^b^	6.66 ± 0.06 ^bc^	6.53 ± 0.06 ^cA^	6.32 ± 0.07 ^dA^
***Overall Acceptability***					
Control	6.89 ± 0.10 ^a^	6.72 ± 0.07 ^a^	6.19 ± 0.07 ^bB^	5.66 ± 0.10 ^cB^	5.25 ± 0.07 ^dB^
BEO-I (T1)	6.95 ± 0.09 ^a^	6.94 ± 0.12 ^a^	6.76 ± 0.08 ^abA^	6.65 ± 0.09 ^bA^	6.56 ± 0.09 ^bA^
BEO-II (T2)	7.05 ± 0.08 ^a^	6.82 ± 0.07 ^b^	6.69 ± 0.07 ^bA^	6.63 ± 0.06 ^bA^	6.39 ± 0.07 ^cA^

Control = No BEO; BEO-I (T1) = 15 ppm bamboo essential oil and BEO-II (T2) = 30 ppm bamboo essential oil. (a–d) mean ± SE with superscript (a–e) on the same row are significantly different (*p* < 0.05). n = 30. (A–B) mean ± SE with superscript (A–B) on the same column are significantly different (*p* < 0.05).

## Data Availability

Data is contained within the article or Supplementary Material.

## Supplementary Materials

The following supporting information can be downloaded at: https://www.mdpi.com/article/10.3390/foods12010218/s1. Table S1: Ingredients used for formulation of meatball emulsion with bamboo essential oil. Figure S1: ZOI formed by different concentrations of BEO against selected pathogenic pure bacterial strains; Figure S2: MIC determination of bacterial strains against BEO using resazurin broth microdilution assay; Figure S3: MBC determination of bacterial strains against BEO using spread plate method after broth microdilution assay.
