# Supplementary material for: Effect of Bamboo Essential Oil on the Oxidative Stability, Microbial Attributes and Sensory Quality of Chicken Meatballs
Source: Foods. 2023 Jan 3;12(1):218. doi: 10.3390/foods12010218 (PMC9819116; doi:10.3390/foods12010218)
Supplement: Supplementary file 1 [file foods-12-00218-s001.zip › foods-2056242-supplementary.pdf]

Supplementary files (S1)

Table S1. Ingredients used for formulation of meatball emulsion with bamboo essential oil

Ingredients (%)	Control	BEO-I (T1)	BEO-II(T2)
Chicken meat	73.5	73.5	73.5
Ice flakes	10	10	10
Refined vegetable oil	7	7	7
Salt	1.6	1.6	1.6
Condiments*	3	3	3
Refined wheat flour	3	3	3
Dry spice mix**	1.8	1.8	1.8
Sodium nitrite (ppm)	150	150	150
Bamboo essential oil (ppm)	0	15	30

Control= No BEO; BEO-I (T1) =15 ppm Bamboo essential oil and BEO-II (T2)= 30 ppm Bamboo essential oil *Condiments: garlic and onion (4:1). **Dry spice mix – aniseed, black pepper, capsicum, caraway seed, cardamom, cinnamon, cloves, coriander powder, cumin seed, turmeric and dried ginger.

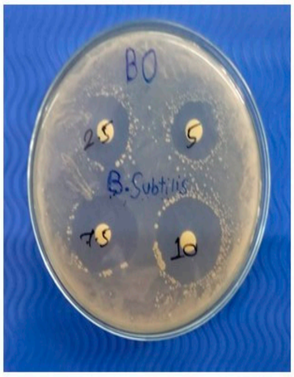

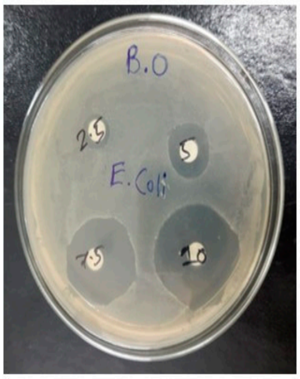

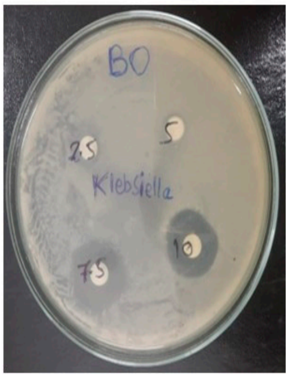

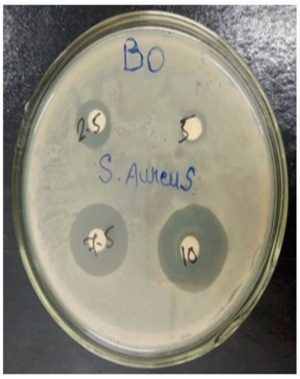

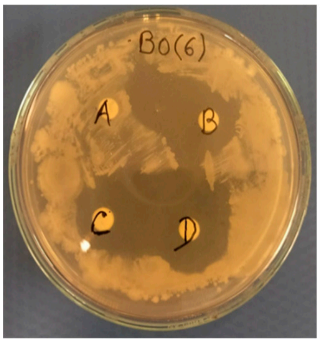

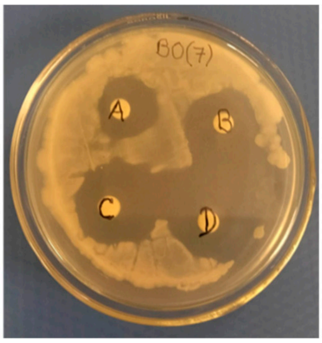

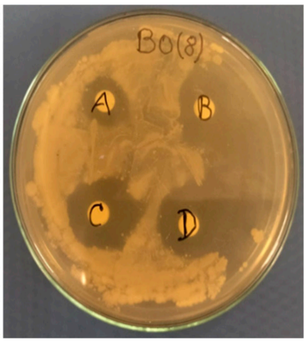

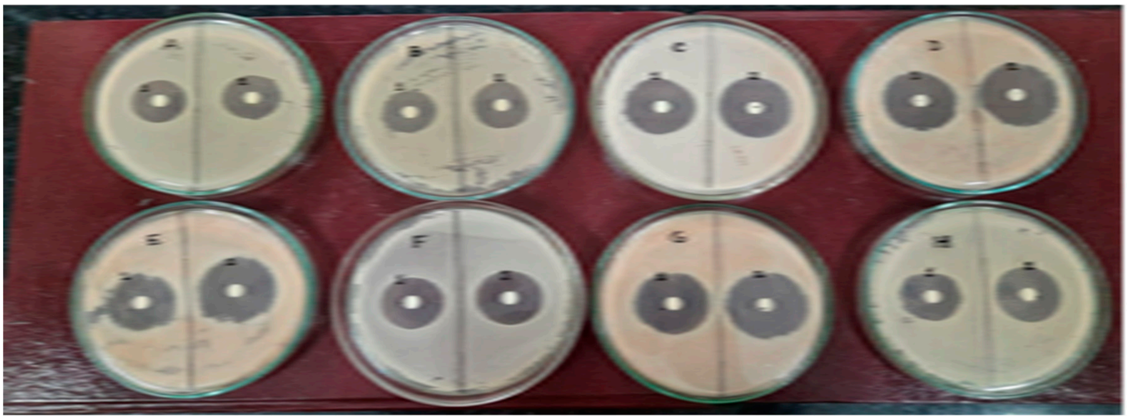

Figure S1: ZOI formed by different concentrations of BEO against selected pathogenic pure bacterial strains

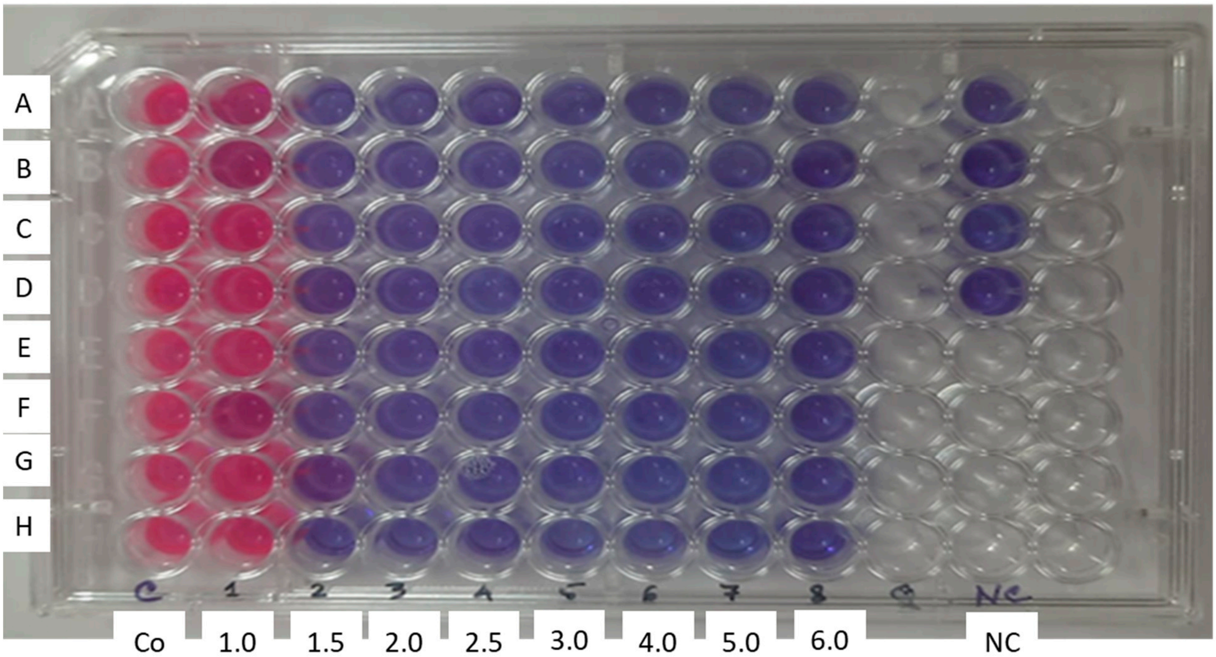

Figure S2: MIC determination of bacterial strains against BEO using resazurin broth microdilution assay

MIC determination of bacterial strains against BEO using resazurin broth microdilution assay. Bacterial strains involved in study: A) *E.coli*, B) *Bacillus Subtilis*, C) *Shigella flexneri* D) *Proteus vulgaris*, E) *Salmonella* Typhimurium, F) *Staphylococcus aureus*, G) *Vibrio cholerae* (0139), H) *Klebsiella pneumonia*. The lower markings show the concentrations of BEO used in each well (µg/µl). NC represents negative control.

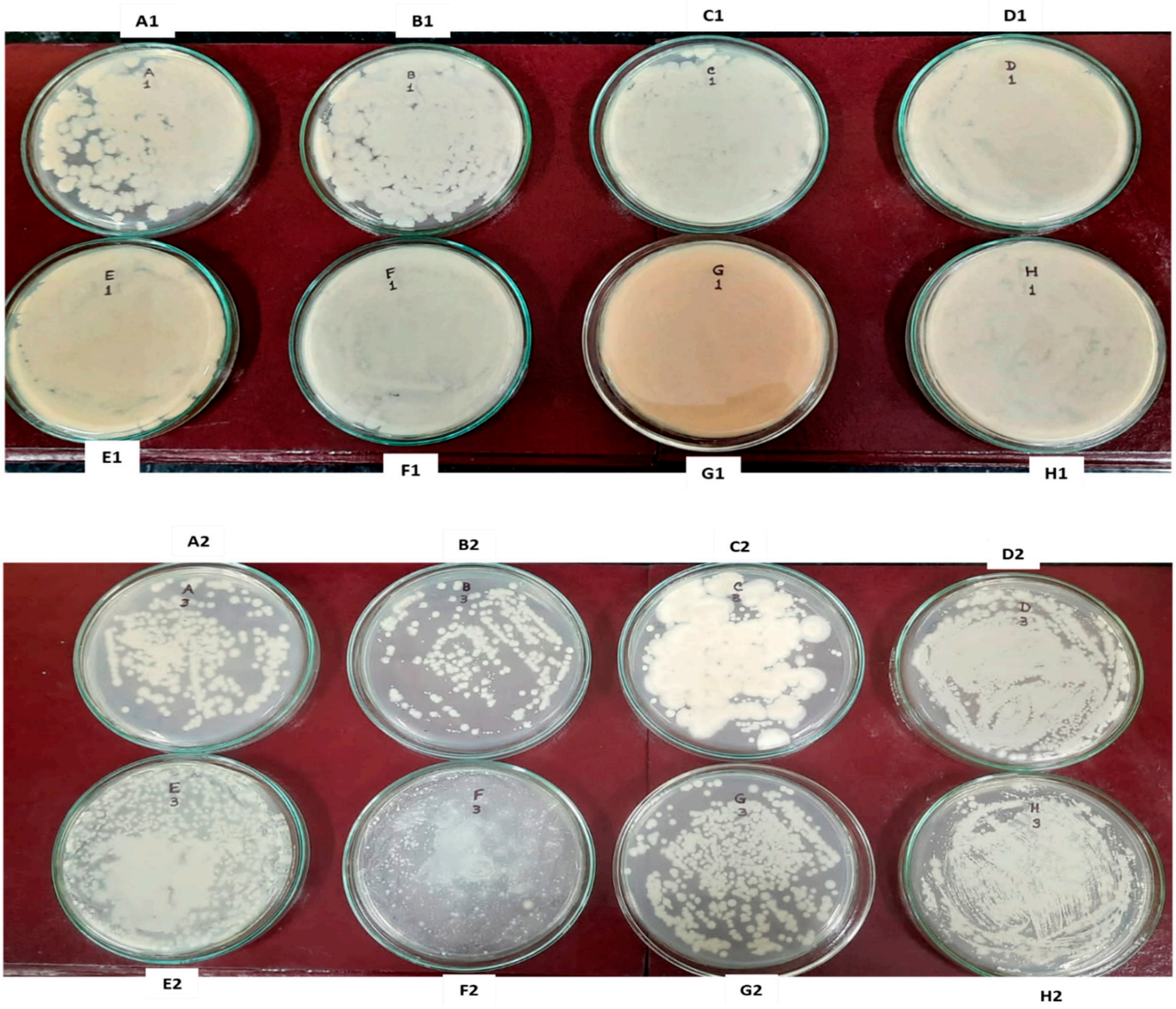
A1
B1
C1
D1
E1
F1
G1
H1
A2
B2
C2
D2
E2
F2
G2
H2

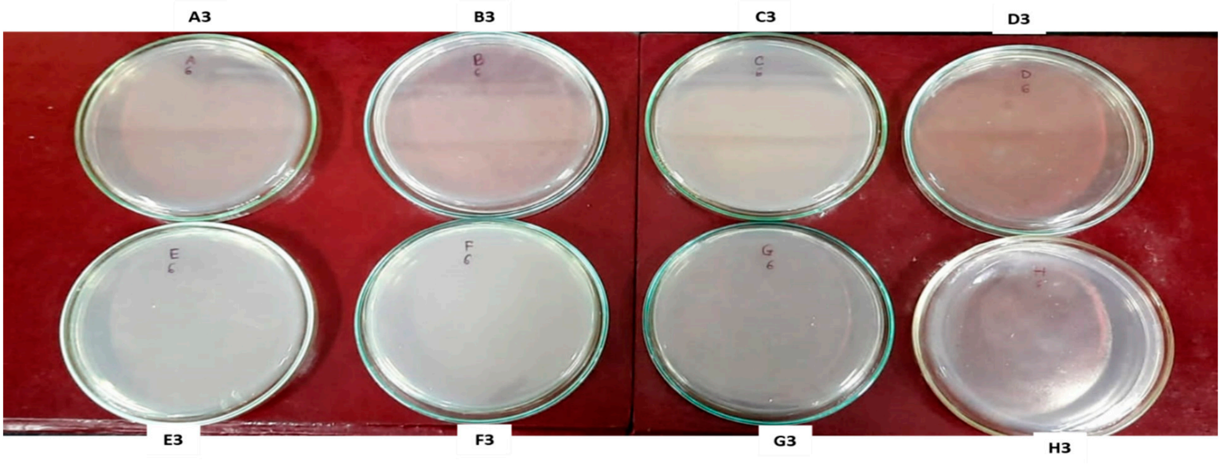

Figure S3: MBC determination of bacterial strains against BEO using spread plate method after broth microdilution assay
